# The Role of Practice Research Networks (PRN) in the Development and Implementation of Evidence: The Northern Improving Access to Psychological Therapies PRN Case Study

**DOI:** 10.1007/s10488-017-0810-5

**Published:** 2017-07-01

**Authors:** Mike Lucock, Michael Barkham, Gillian Donohoe, Stephen Kellett, Dean McMillan, Sarah Mullaney, Andrew Sainty, David Saxon, Richard Thwaites, Jaime Delgadillo

**Affiliations:** 1 University of Huddersfield, Harold Wilson Building, Queensgate, Huddersfield, HD1 3DH UK; 2 South West Yorkshire Partnership NHS Foundation Trust, Wakefield, UK; 3 Clinical Psychology Unit, Centre for Psychological Services Research, University of Sheffield, Sheffield, UK; 4 Sheffield Health & Social Care NHS Foundation Trust, Sheffield, UK; 5 Department of Psychology, University of Sheffield and Sheffield Health & Social Care NHS Foundation Trust, Sheffield, UK; 6 Department of Health Sciences, University of York, York, UK; 7 Humber NHS Foundation Trust, Hull, UK; 8 Centre for Psychological Services Research, University of Sheffield, Sheffield, UK; 9 Cumbria Partnership NHS Foundation Trust, Penrith, UK; 10 Clinical Psychology Unit, Department of Psychology, University of Sheffield, Sheffield, UK

**Keywords:** Implementation science, Practice research network, Psychological therapies, Outcome monitoring

## Abstract

Practice research networks (PRNs) can support the implementation of evidence based practice in routine services and generate practice based evidence. This paper describes the structure, processes and learning from a new PRN in the Improving Access to Psychological Therapies programme in England, in relation to an implementation framework and using one study as a case example. Challenges related to: ethics and governance processes; communications with multiple stakeholders; competing time pressures and linking outcome data. Enablers included: early tangible outputs and impact; a collaborative approach; engaging with local research leads; clarity of processes; effective dissemination; and committed leadership.

## Introduction

Implementation science provides a structure to study the challenges and barriers of translating evidence based practice (EBP) into routine healthcare in a timely way (Proctor et al. 2009). Tansella and Thornicroft (2009) suggest that the translation of clinical guidelines into routine clinical practice and the reduction of inappropriate variability in practice are important implementation challenges for mental health services. To address these challenges in mental health, it is important to understand how contextual factors influence practice and to use a participatory approach to develop strategies for implementing relatively complex interventions in community settings (e.g. Mendel et al. 2008). Practice Research Networks (PRNs) could support such an approach by enabling partnerships between practitioners and researchers, thereby linking the realities of routine care with the methodological rigor required to successfully understand and overcome implementation issues.

PRNs can also support the generation of practice-based evidence (PBE) as an important complement to EBP and trials methodology (Barkham and Margison 2007; Barkham et al. 2010; Castonguay et al. 2013). PBE comprises evidence on the provision of interventions in routine practice, ideally along with contextual information to understand some of the local implementation issues. Practice-based datasets can provide larger sample sizes than typical efficacy studies and support more clinically meaningful research without the exclusion criteria often found in randomised controlled trials. Practice-based studies can also enable the benchmarking of clinical outcomes, comparing services with one another and comparing routine practice effects with those observed in efficacy studies (Barkham et al. 2008). It is, however, important to take account of the limitations of routinely collected outcomes data, such as the implications of missing data, potential biases in the data collected and over-emphasising uncontrolled treatment effects (Clark et al. 2008).

The aim of the present paper is to describe the processes involved in setting up a practice research network (PRN) involving a number of Improving Access to Psychological Therapies (IAPT) services in the North of England, together with impact on services. We will provide some background on the history and role of PRNs, describe the IAPT program and highlight the opportunities and challenges it presents in setting up a PRN. We will then describe the setup and early development of the PRN, including its structure, membership, and development, with reference to the Exploration, Preparation, Implementation and Sustainment (EPIS) framework (Aarons et al. 2011). The EPIS framework has been chosen as an implementation framework because it was developed to be applicable to public services and acknowledges the importance of local contexts, both within organizations and in the wider environment. It proposes four implementation phases, Exploration, Preparation, Implementation, and Sustainment and the last two are particularly relevant to the IAPT PRN which takes advantage of the data generated by an existing program and uses this to contribute to effective implementation.

We will identify and reflect on some of the issues, challenges, and opportunities experienced by the PRN in its development phase, focussing on one study as a case example. Finally, we will discuss the early impact on practice and implications for developing effective PRNs in the future.

## Practice Research Networks (PRNs)

PRNs involve the active engagement of practitioners and researchers in partnership to provide a vehicle through which research evidence can be implemented. They also enable research to be grounded in the priorities of clinical services, supporting reflective practice and resulting in a more immediate impact upon practice. PRNs have their origins in medicine with the pioneering work of key individuals including British General Practitioners James McKenzie, Will Pickles, and John Fry (see Barkham 2014; Green and Hickner 2006). They were widely adopted in the US as a means for capturing data from community settings, particularly family physicians, with the Ambulatory Sentinel Practice Network marking a watershed (Green et al. 1984) and have endured and grown over time (Hickner and Green 2015).

In psychotherapy research, some PRNs have been developed within types of therapy (e.g. Huet et al. 2014; Andrews et al. 2011) and others which involve the use of common outcome measures across several psychotherapy services. An example of the use of a common outcome measure is the Pennsylvania Psychological Association Practice Research Network (PPA-PRN) which used a core assessment battery to collect pre-and post outcomes data to support practice-based research (Borkovec et al. 2001; Castonguay et al. 2010). Barkham (2014) suggests various PRN models or features which reflect the diversity of the approach: a grass roots model (e.g. Huet et al. 2014); a developmental model in which clinicians and researchers build up the use of clinical tools in a planned way (Sales et al. 2014); a center model with a single site (e.g. McAleavey et al. 2014); and a star model with multiple sites (e.g. Cooper et al. 2014). Despite the widely acknowledged benefits of PRNs, they remain under-utilized in mental health settings (McMillen et al. 2009). The limited adoption and impact of PRNs in mental health settings in countries such as the UK (e.g., Audin et al. 2001) is likely to be due to challenges and barriers such as funding, sustainability, scientific outputs, and impact (McMillen et al. 2009). McMillen et al. (2009) also identify potential barriers, which include managing relationships within the PRN, productivity, ethical and governance arrangements.

## The Improving Access to Psychological Therapies (IAPT) Programme

The IAPT programme in England has radically changed the way therapies are organised and delivered (Clark 2011; Layard et al. 2007). The programme began in 2006 with two demonstration sites offering evidence based psychological interventions to adults of working age with depression and anxiety problems. The programme later expanded over the next decade, so all areas in England now have IAPT services. IAPT is grounded in a stepped care service model (Bower and Gilbody 2005), offering access to low and high intensity interventions according to need and patients’ response to entry-level low intensity interventions. Step 1 usually involves contact with a general practitioner for assessment, advice and medication. Step 2 comprises brief, low intensity interventions, such as guided self-help, that are provided for mild to moderate depression and some anxiety disorders, while step 3 comprises protocol-driven high intensity psychological therapies including cognitive-behaviour therapy (CBT), interpersonal psychotherapy, counselling for depression, behavioural couples therapy, eye-movement desensitisation and reprocessing (EMDR), and brief dynamic interpersonal therapy. Gyani et al. (2011) report from the first year of IAPT data that a median of 28% of patients were stepped up to step 3 interventions, with a great deal of variation between services. Implementation of EBP is at the heart of the IAPT programme, which is informed by the National Institute for Health and Care Excellence (NICE) guidelines for depression (2009) and anxiety (2011). Indeed, the case for the investment in IAPT was based on the need to implement NICE guidance as well as an economic case that the investment would help to reduce welfare benefits and medical costs associated with mental health related disability (Layard et al. 2007).

A significant achievement of the IAPT program has been the use of common outcome measures across all services (see IAPT National Programme Team 2011). Outcomes are measured at each session using the Patient Health Questionnaire (PHQ-9; Kroenke and Spitzer 2002), a measure of depression; the Generalised Anxiety Disorder Assessment (GAD-7; Spitzer et al. 2006); and the Work and Social Adjustment Scale (WSAS; Mundt et al. 2002). Measures are gathered from patients at every contact, with completion rates of up to 90% (Department of Health 2011a). The scale and achievements of the IAPT programme have been impressive, with more than one million people using the service in the first 3 years of implementation. Approximately 683,000 patients had completed a course of treatment during that time, with recovery rates in the region of 45% (Department of Health 2011a). The most recent figures available for the IAPT programme showed that for the year from 1 April 2015 to 31 March 2016 1,399,088 million new referrals were received, of which 953,522 (68%) entered treatment and 537,131 (38%) finished a course of treatment (Health and Social Care Information Centre 2016). IAPT service providers generate data on the delivery and outcomes and this shows that reliable recovery rates vary considerably between services (Gyani et al. 2013). The Northern IAPT PRN represents approximately 10% of the overall IAPT service provision, based on the proportion of localities involved.

IAPT has also attracted its critics. For example, Guy et al. (2012) criticise the approach taken by NICE guidance and McPherson et al. (2009) argue that NICE guidance is based on studies using symptom-based outcome measures rather than measures of functioning or quality of life. Scanlon (2015) criticises the emphasis on return to work, and argues that IAPT encourages a dialogue that emphasises individuals’ psychological failures rather than the consequences of national and international economic policies.

The use of routine measurement of clinical outcomes in IAPT services has led to a large volume of research on outcomes in routine practice. Data from the initial demonstration sites have led to publications on clinical outcomes (Clark et al. 2009; Richards and Borglin 2011), attendance (Di Bona et al. 2014), cost-effectiveness (Mukuria et al. 2013) and the wider impact of this service delivery model on hospital admissions, use of antidepressants and return to work (de Lusignan et al. 2012, 2013). Further multi-site IAPT datasets have been used to report service-level performance indicators (e.g. see Health and Social Care Information Centre 2015), as well as to benchmark clinical outcomes (Delgadillo et al. 2014a; Glover et al. 2010; Royal College of Psychiatrists 2011, 2013) and to explore the determinants of variability between low intensity practitioners (Green et al. 2014; Firth et al. 2015) and services (Delgadillo et al. 2016a; Gyani et al. 2011).

Other studies using single-service level IAPT datasets have also been published in recent years (e.g., Ali et al. 2014; Böhnke et al. 2014; Burns et al. 2016; Cairns 2014; Delgadillo et al. 2014b, 2016c; Goddard et al. 2015; Saxon et al. 2016). However, single-service datasets are often too small for the study of particular clinical groups (e.g., specific ethnic minorities or patients in particular socioeconomic situations) or for the study of therapist and service level effects. The potential yield from PRNs and large datasets is also facilitated and enhanced by the growth of advanced statistical analytic procedures designed to account for the hierarchical nature of service delivery data (i.e., patients nested within therapists nested within teams nested within services). This requires the establishment of collaborations between academics and service providers, which enable suitably powered analyses of data from multi-service networks.

The standardisation of EBP in IAPT services and large and growing datasets provide opportunities to build connections between researchers and clinicians to generate practice-based evidence relevant to practitioners, services, commissioners and policy makers and to understand implementation issues in routine practice. The importance of implementation is underlined by the variations in outcomes and other performance indicators across services (Gyani et al. 2013). The limited funding within services to support research is a challenge to this aspiration and we argue that one realistic and effective way of delivering this is through PRNs.

## The Northern IAPT PRN: Organisational Infrastructure

### Setting up the Northern IAPT PRN

The origins for the Northern IAPT PRN lay in a shared vision and informal discussions held at an international conference (Society for Psychotherapy Research, Copenhagen 2014) between researchers from the northern Universities of York, Huddersfield, and Sheffield. From this starting point, an expression of interest and invitation was mailed to IAPT services across the Yorkshire and Humber and North West regions of England to an initial open meeting in September 2014 to discuss the potential development of a PRN. The meeting comprised representatives of nine IAPT services and the three universities. The meeting agenda included an explanation of the concept and purpose of a PRN, rationale for a PRN across IAPT services, and also discussions of the mission, scope, and structure of an IAPT PRN. A key rationale for setting up the PRN was to utilize the data available from routine outcomes monitoring for research and the improvement of clinical services. It was agreed that the PRN could utilise existing datasets to contribute to generating practice-based evidence on the delivery of psychological therapies in routine services. The initial meeting also emphasised the non-competing reciprocal needs of both researchers and services.

Current or proposed collaborative research projects involving potential members were presented with posters and briefings to demonstrate current collaborations, and potential sources of research funding were also discussed. The decision to form a PRN was agreed, a Chair elected, and a draft mission and scope document was subsequently co-produced through email discussions (see summary in Table 1). A key decision at that time was to focus on two projects that already had clear momentum and were likely to lead to success in the first year: (a) A multi-site study of the effectiveness stress control (SC) classes. The stress control study is described in more detail below and used as a case example to reflect on the work of the PRN in terms of implementation issues and impact. We have chosen this study because of the challenges it presented and the tangible impacts achieved; (b) A transdiagnostic seminars project which involved offering access to a series of three psychoeducational seminars prior to starting formal cognitive behavioural therapy (CBT). This followed previous research by members of the PRN which suggested that the seminars resulted in greater treatment completion rates compared to usual CBT (without access to seminars) and therefore enhanced engagement and reduced dropout in formal high intensity CBT (Delgadillo and Groom 2017). The choice of these initial projects was determined by consensus and based on their likelihood of achieving tangible successes in the first year.

**Table 1:** Mission and scope of Northern IAPT Practice Research Network

Mission
The network’s mission is to generate practice-based evidence that will influence psychological services in England
The network’s aspiration is to develop a research culture within its constituent services. It will do so through a ‘bottom-up’ process of research carried out in the front line of mental healthcare
The network will operate independently from the central IAPT programme or Department of Health, but will forge links with key stakeholders, funding bodies and policy makers in its goal to influence practice
Scope
The network will start by including northern IAPT services/universities, but would be open to including other interested partners in the future
The network will embrace both quantitative and qualitative research methods
The network will primarily focus on learning from retrospective clinical data collected by its constituent services, but will also consider projects that involve prospective data collection

### Organisation and Operation of the PRN

An operational plan was agreed which included specific guidelines on project development and management, shared working, communication, and dissemination. A key understanding was that the PRN was both a collective and collaboration and was not led by any one organisation. Information has been shared with PRN members via regular emails and monthly progress reports have been made available on the website (http://www.iaptprn.com), as well as through regular emails to network members and communications through social media sites like Twitter and professional membership bulletins (e.g. *Therapy Today, CBT Today*). IAPT services and academic institutions can opt-in as network members through a formal process which involves four steps: (1) making contact via the PRN website to express interest in joining; (2) identifying a named person who will be a representative of the joining organisation; (3) downloading and reading the PRN terms of reference document; (4) completing and submitting a formal membership application to the PRN chair. By the end of 2015, 14 IAPT services and six universities had joined the network through this process.

A flexible way of operating was agreed whereby IAPT services would ‘opt in’ to projects they judged to be a priority and feasible, although progress on these projects would be shared with the whole network. All projects would have teams which included representatives from each participating service (ascribed as a principal investigator for the purposes of NHS ethics and management approvals) and the relevant academic research support. Each study would also have a project lead responsible for the overall management of the project, ethical approvals, information governance and organisational permissions. Project team members had clear roles depending on their service role and/or expertise. The PRN provides virtual and actual forums to enable project teams to meet and share ideas, report the progress of ongoing projects and offers support in terms of methodology and funding streams. PRN steering group meetings provide a formal mechanism to review progress, which representatives from all services could attend, and the main functions are to: (1) enable project teams to meet in person, (2) communicate progress to the wider PRN members, (3) obtain advice and support from the wider PRN members to advance current projects, and (4) scope future opportunities for funding/grant applications.

Another function of the PRN is to disseminate research findings to key stakeholders including front-line clinicians, service users, service managers, and professional organizations such as the national IAPT programme, British Association of Behavioural and Cognitive Psychotherapies (BABCP) and British Association for Counselling and Psychotherapy (BACP) in order to influence policy and practice. The PRN sought to inform these stakeholders through its multiple modes of communication (webpage, social media posts, email newsletters) as well as through more formal peer reviewed publications, national conference presentations and regional CPD events for IAPT staff groups. Aligned to its goal of influencing policy and practice, the PRN has also published a series of open-access ‘debate’ articles during 2015, along with electronic surveys and a ‘blog’ facility to engage the wider public in discussing current and provocative aspects of clinical practice in IAPT services. The debate articles comment on workforce issues such as commissioning models (Sainty 2015), staff retention and turnover (Moreea 2015), professional registration (Kell and Kay 2015), and clinical issues such as the challenges raised by social deprivation (White 2015). During the first year, the PRN website content was accessed 9759 times by 4020 unique users. The average number of participants that responded to public surveys was 57 (range 48–63); these were predominantly psychological therapists and managers working in IAPT services, though the surveys were also completed by members of the general public and service users.

## Case Example: The Stress Control study

The stress control study was chosen as a case example because it was one of two projects supported by the PRN in the first year and met with a wide range of challenges which highlight implementation issues. Stress control is a six session CBT-based psycho-educational class, provided to large groups of patients in community settings (White et al. 1992). It is the most widely used and validated stress management course provided in the NHS and in England they are provided by IAPT services. It is delivered as an educational class, often in local community venues, sometimes to groups of over 100, with no discussion of personal problems, nor facilitation of group discussions and mutual support. Stress control is therefore evidence-based in that it is a CBT intervention, but the different mode of delivery means it is important to generate more evidence on its provision and effectiveness in routine practice.

Stress control classes have been provided to people with a wide range of problems, including anxiety, depression, panic, insomnia, poor self-esteem, low self-confidence, irritability and anger, drinking, using drugs to excess, and burnout. Typically sessions are weekly and last for about 90 min with a short break. There is some evidence that Stress control is effective at engaging “hard to reach” groups such as men, ethnic minorities and those from more deprived areas and that outcomes are comparable to individual therapy (White et al. 1992; Woodet al. 2005). This raises the possibility that it may be more efficient than individual therapy given the high volume, low intensity nature of the intervention (Kellett et al. 2007). As stress control is no panacea, it is important to develop a better understanding of who the intervention works best for, the most effective delivery methods and how it should fit in to stepped care.

The aims of the stress control study were: (1) to describe the current provision of SC interventions across several IAPT services in the north of England; and (2) to assess whether it is possible to identify patient characteristics associated with attendance and clinical outcomes. The method involved aggregation of retrospective data for IAPT patients who have accessed SC interventions over a 2-year period of time. Descriptive statistics were used to profile outcomes as a function of sessions attended and multilevel regression modeling was used to investigate predictors of clinical outcomes defined by depression (PHQ-9, Kroenke and Spitzer 2002) and anxiety (GAD-7; Spitzer et al. 2006) measures. This study is reported in full elsewhere (Delgadillo et al. 2016b).

We will highlight what we have learned from the development of the PRN in terms of feasibility, processes, pragmatic solutions and impacts using the stress control study as a case example. Table 2 shows the timeline for key milestones for the PRN and the stress control case study.

**Table 2:** Timeline of key achievements for the PRN and stress control study in the first 2 years

Date	PRN infrastructure and organization	Stress control (SC) study
September 2014	The initial PRN set up meeting for the Northern IAPT PRN	
	Representatives of 9 IAPT services and 3 universities attended	
	Decision to focus on 2 projects: (1) Stress control (SC); and (2) Transdiagnostic seminars (TS)	
October 2014		Research grant secured for SC study
November 2014	Northern IAPT PRN website launched: http://www.iaptprn.com	The initial project team meeting for the SC study Briefing paper produced to update collaborating services about SC study
January 2015	Presentation about Northern IAPT PRN delivered at the IAPT North West Leadership and Innovation Forum, Preston	Integrated Research Application System (IRAS) form prepared to apply for NHS ethical approval
February 2015		NHS Research Ethics approval obtained within 3 weeks of the application
March 2015	The *Debate* webpage launched to promote public discussion about current IAPT policy and practice. http://www.iaptprn.com/debate Twitter account set up: @iapt_prn	Completed scoping questionnaires requested from participating services
April 2015		Information sharing agreement provided, to be signed by the relevant individual in each organization Principal Investigators at each research site asked to liaise with local IAPT service data leads and their R&D departments Formal request for data from each service in Microsoft Excel compatible format on all cases that had contact with the IAPT service and were discharged between 01/01/2013 and 01/01/2015
May 2015	The public survey results made available on website for first *Debate* article on topic of commissioning models for IAPT services New article and survey published covering topic of insufficient supply and rapid turnover of the Psychological Wellbeing Practitioners (PWP) workforce Annual PRN meeting held 28th May. Representatives from 7 IAPT services and 4 universities attended to discuss future research priorities	R&D management approvals received from eight organizations providing IAPT services for permission to host the study Information sharing agreement provided by services Datasets provided over the next 2 months
June 2015	The PRN website received >1700 unique visitors in past 6 months Site attracted approx. 500 new users since the launch of the *Debate* webpage The *Debate* webpage redesigned including a ‘blog’ feature to allow open comments about the article topics	Datasets received from five IAPT services provided by four NHS Trusts
August 2015	New *Debate* article published covering topic of professional registration of Psychological Wellbeing Practitioners (PWP) workforce Public survey results for the first *Debate* article published online	Data aggregation and cleaning Initial descriptive analysis
September 2015	PRN promoted in *Therapy Today* (British Association of Counselling and Psychotherapy) and *CBT Today* (Behavioral and Cognitive Psychotherapy) Public survey results for the second *Debate* article published online	Presentation of initial results at meeting of SC collaborators Agreed plans for publications on SC project Identified specific grant capture and associated application
November 2015	New *Debate* article and survey published discussing the relationship between poverty and mental healthcare Public survey results for the third *Debate* article published online	Concluded and disseminated a study outcomes report for funders and collaborating services Submitted study manuscript for peer review in a scientific journal, with author list including academic and clinical members of the PRN
February 2016	Public survey results for the third *Debate* article published online	Consultation with a group of patients who accessed stress control interventions in participating IAPT services Developed a summary of patients’ perspectives and recommendations in relation to stress control interventions delivered in routine care
June 2016	Annual Northern IAPT PRN meeting held at the University of Manchester	Findings of the stress control study presented at the British Association for Behavioural and Cognitive Psychotherapies (BABCP) annual conference Findings discussed and future recommendations for routine clinical services co-produced at the annual Northern IAPT PRN meeting
July 2016	Public survey results for the fourth *Debate* article published online	
September 2016		Stress control research manuscript accepted for publication in scientific journal Behavior Research and Therapy, doi: 10.1016/j.brat.2016.09.010
October 2016		Recommendations for routine IAPT services delivering stress control interventions published in the PRN website, promoted by social media channels and disseminated to all member organizations

### Ethical and Service Management Approvals

The analysis of anonymised and aggregated datasets is likely to be a significant part of the future work of the PRN. This was the approach taken in the stress control study and we will describe and reflect on our experience of the ethical considerations and NHS management approvals required for such research. Researchers in the UK need to consider a number of options regarding the ethical review and governance of healthcare research, in particular that which includes data from NHS patients. In simple terms, current guidelines outline different levels of permissions required for different types of studies (Department of Health 2011b). The levels progress from (1) no requirement of ethical review for service evaluations and audits using anonymized data; to (2) an expedited ‘proportionate review’ by an NHS ethics committee for formal research that does not carry significant ethical issues; and finally (3) full ethical review by an independent NHS ethics committee for formal research which carries significant ethical issues. Although ethical approval is not mandatory for studies which rely on secondary analyses of routinely collected clinical data, as long as it is strictly de-identified (Department of Health 2011b), independent review and scrutiny by a research ethics committee is good practice and journals often require clear information regarding ethical approval. The proportionate review process (option 2) was adequate to enable the PRN to carry out secondary analyses of routinely collected and anonymous clinical datasets.

Obtaining service management approvals from the organizations hosting the study was a lengthier and more complicated process and involved liaising with nine IAPT services within eight UK NHS Trusts. The need to obtain approvals from several management and administrative personnel in each site has the potential to substantially delay the commencement of studies and an effective strategy we used to streamline communications and permissions was to have named principal investigators (PI) in each participating IAPT service. The local PIs acted as brokers between the wider research team and the NHS personnel who needed to be involved in the formal permissions to approve the study (e.g. research and development (R&D) staff, information governance staff, information analysts, Directors responsible for R&D and heads of service), and in the preparation of datasets (information technology staff). This arrangement also made it clear to the NHS research ethics committee that the research was collaborative, with good engagement of services. The local PIs also supported the research team in recruiting patients for our Public and Patient Involvement (PPI) work stream, and communicated the study updates and results to clinicians and managers in their IAPT services, important for dissemination and impact. We also provided a short project briefing to help PIs inform local services and practitioners about the study and provided a data sharing agreement so it was clear to each organization what the data would be used for. It was made clear that analysis outside the study protocol would require further approvals.

### Public and Patient Involvement (PPI)

The importance of public and patient involvement (PPI) in research is well established (Brett et al. 2010). During its first year, the approach taken by the PRN was to consider PPI in terms of individual studies rather than representation on the PRN as a whole. For example, one of the IAPT services involved in the stress control study recruited patients who had attended stress control classes. The findings of the study have been fed back to them and their views sought. These views have provided a valuable perspective on the findings and of their experiences of attending stress control classes, which have led to the identification of service improvements and possible further research. The aim is for this group of patients to work with the project team to co-produce recommendations and guidance for stress control classes in the future. The study findings and patients’ views were presented at the most recent annual meeting of the PRN (June 2016). These were then discussed and informed network members’ recommendations for clinical practice, disseminated in October 2016.

### Data Collection, Preparation and Linkage

An important challenge to the accomplishment of the goals of the PRN concerns the gathering and standardization of routinely collected datasets. The role of the data manager in each service is vital in the functioning of the PRN and the progress of various projects. For the stress control study, in order to be able to link datasets from multiple services, we developed a standard dataset specification document, which described all of the variables necessary to accomplish each of our intended data analyses. The specification document was provided to information technology staff at participating IAPT services, to ensure data was aggregated in a standard format. This close service liaison enabled us to identify and resolve discrepancies in how different services ‘code’ specific data fields. For example, some services had a specific label to identify attendance at ‘stress control’, whereas other services used a generic label for ‘group psycho-education.’ This made it difficult to distinguish stress control from other group based psychoeducational interventions. In our experience, a key challenge to the adequate linkage of datasets is posed by the fact that different services use different clinical data management systems and also invest to a different degree in the role of a data manager (i.e. some services lacked a data manager). Ultimately, we found that some clinical data management systems are inadequate for data mining purposes, and this was a key problem that meant that we were unable to extract data from two IAPT services. A dedicated data manager is clearly recommended.

In summary, our experience over the first year of operation was that for the stress control study it was possible to obtain datasets and conduct research with five out of nine IAPT services that initially signed up to work with the PRN. The main obstacles preventing participation from four services related to competing pressures on the time of some local PIs and the inadequacy of some clinical data management systems which did not allow us to collect the level of standardized data necessary for our intended analyses.

### Impact on Practice and Further Research

The impact of the stress control study on routine practice was reviewed in September 2016, 2 years after the study began, and less than 1 year after the study was completed. The findings have been fed back to services, including the SC facilitators. Recommendations for routine IAPT services delivering stress control interventions were published on the PRN website and disseminated to all member organizations in October 2016. These include the importance of adhering to the recommended six session format, suggestions for engaging clients to attend all sessions (such as presenting our outcome data), and providing more personalised and/or intensive treatment options for those who did not benefit. Impacts up to that time include: one service who changed their provision from a five session format to the six session format; greater emphasis on inviting friends and family members to the classes and acknowledging their role; more focus on training in presentation skills. Other services concluded that their provision of the six session format was validated by the study. Further planned service developments include developing a modified stress control programme for clients in recovery from substance misuse issues in conjunction with the local drug service and applying for funding to co-develop a version for university students. In addition to being disseminated through a peer reviewed publication (Delgadillo et al. 2016b), the stress control study was also presented at a symposium the British Association of Behavioural and Cognitive Psychotherapy conference in June 2016, chaired by the originator of the SC program, Jim White, which included papers from Northern Ireland, the Republic of Ireland, Wales, and Belgium, thereby broadening dissemination and future research collaborations.

## Discussion

This paper has described the setup, processes, and work of the Northern IAPT PRN in its first year of operation using one research study as a case example. In terms of the role of the PRN in implementing EBP, we will consider the EPIS framework (Aarons et al. 2011). The IAPT programme was set up to implement EBP, in the form of UK NICE guidance, and reducing inappropriate variability in practice has been identified by Tansella and Thornicroft (2009) as a key implementation challenge in mental health. In terms of the four phases of the EPIS framework, Exploration, Preparation, Implementation, and Sustainment, the PRN capitalizes on a program that has already been set up, so the IAPT PRN is more pertinent to the implementation and sustainability phases. Implementation can be supported by a PRN through collating and benchmarking routine outcomes across services, investigating factors influencing variability and improving outcomes across services. The EPIS framework also highlights the importance of inner and outer context which we found was crucial in understanding how the SC intervention was provided. There was variability in terms of outer context such as urban versus rural settings and arrangements necessary to improve access. Other outer contextual factors include the government support to set up and fund the IAPT program, continued Department of Health and policy support, agreements with commissioners, availability of appropriate settings to provide the SC classes, and how SC fitted into the local stepped care service model. Inner contextual factors found to be important included the style and confidence of facilitators, variation in terms of fidelity to the recommended SC model and variable practices in terms of the involvement of partners, friends and family members. The EPIS framework also highlights the importance of organizational culture (Glisson and Williams 2015) in innovation, service quality and outcomes. We believe that participation in a PRN is likely to support a culture of reflective practice that supports these aspirations.

It is already clear to those of us involved that a PRN provides a very good model for establishing collaborations between service providers and academic researchers. A number of lessons have been learned along the way which we now discuss. Reciprocity is clearly at the heart of PRN work. However, carrying out collaborative research under the PRN model comes with challenges related to (a) research ethics and governance processes, (b) co-ordination of communications with multiple stakeholders, (c) competing time pressures on PRN members, and (d) technical difficulties in gathering and linking data from different clinical data management systems. Mindful of these obstacles, we have identified a series of inner contextual factors that have facilitated the establishment and success of the Northern IAPT PRN.

1. The PRN comprised researchers with a historical and shared research vision and expertise in collecting and analysing large routine datasets prior to the national IAPT initiative (e.g., Barkham et al. 2001; Lucock et al. 2003). Critically, this is not a *sine qua non* for setting up a PRN. However, having a long held vision and experience in working with large psychological therapy datasets provided impetus and support.
2. A committed academic chair of the PRN (JD) has proved to be a driving force for the network. Similarly, having a core group of committed academics who actively drive the development of key outputs (e.g. research manuscripts, grant applications, social media communications) has been invaluable to keep a momentum of work. These aspects, which can be summarised as (a) funding, (b) commitment and (c) momentum, are consistent with the recommendations of other research groups that have noticed that productivity is essential to the survival and profile of PRNs (McMillen et al. 2009).
3. The primary reliance on a research strategy focusing on large routinely collected data requires ready access to statistical expertise with skills in both traditional statistics but also multilevel modeling, skills that the PRN is fortunate to hold.
4. Academic and clinical members of the PRN came to a consensus agreement about the focus and objectives of initial projects. This ensured that the research conducted by the PRN was of clear relevance to clinical collaborators, which is a fundamental incentive for them to remain involved. Unsurprisingly, the pressures and demands of routine clinical care and performance targets can impede clinicians and service managers from working with the PRN. It is therefore crucial that PRN studies closely align to the interests and priorities of clinicians and services in order to maximise participation, engender a sense of ownership (Tasca et al. 2015) and provide a justification for involvement.
5. Achievable targets in the initial year helped to galvanise support in working collaboratively and also reinforced PRN members’ commitment once the tangible outputs of the initial studies were achieved and communicated.
6. Robust collaborations with leading practitioners and managers as well as experienced academic researchers helped to bolster the credibility of the PRN, as well as the commitment of its members. Enlisting members who have peer influence can be an important means of enhancing pro-research social norms and attitudes within clinical services, thus increasing their likelihood of participation in PRN activities (Tasca et al. 2014).
7. The open, welcoming and collaborative ethos of the PRN enabled a forum for sharing ideas and good practice. A barrier to this was too much research jargon, which can be off putting to clinicians, so it is important to pitch discussions to engage all parties.
8. Having clear and formal operational processes helped to successfully co-ordinate communications, to obtain approvals and to gather and analyse routine clinical data within the scope of specific projects. The challenges we faced in obtaining permissions from multiple stakeholders and governance bodies are by no means unique to research in IAPT or NHS services (e.g. see Wolf et al. 2002).
9. Having an online presence through the PRN website and social media campaigns and communications helped to raise the public profile and increase the membership of the network.
10. Outputs from the PRN accurately and fairly reflect the work conducted by the various members of the PRN; this includes the recognition of clinical collaborators as co-authors of peer reviewed papers.

These factors helped our network to overcome the common challenges for PRN-based research and are linked to the following recommendations: a collaborative approach to identifying research topics; engaging with local PIs for specific projects; committed leadership for both the PRN and participating organisations; identifying projects which are feasible and can deliver tangible outputs in the first year. The PRN is at an early stage, but we believe it has potential to support high quality research to further the aspirations of the IAPT programme which include improved access, clinical improvement and recovery, improved social and economic participation, and increased patient choice and satisfaction (Department of Health 2011c). An effective PRN can also contribute to the wider psychotherapy outcomes research and provide a vehicle through which barriers to the implementation of EBP in psychological therapies, highlighted by Shafran et al. (2009), may be overcome. The alignment of the activity of the PRN with the business plans and associated targets of the services facilitates organisational commitment to the research process. The involvement of researchers ensures that the limitations of routinely collected data, including missing data and uncontrolled treatment effects, are acknowledged and factored into analyses and the conclusions that can be drawn.

A key element of the rationale for setting up the PRN was the opportunity to make use of historical data collected from routine outcomes monitoring across different services using the same outcome measures. We have shown that this is indeed possible but not without challenges and we hope this paper will help others anticipate and overcome the challenges. Making use of routinely collected outcome and service data for research also adds to the justification for the time patients and clinicians spend on data collection. There is clearly huge potential for PRNs to contribute to generating practice-based evidence on many aspects of the delivery of psychological therapies in routine services and our experience shows what can be achieved with good will, commitment, leadership, a genuine sense of collaboration between academics and clinicians (McMillen et al. 2009) and some research funding.
